# Interplay between DNA and RNA Modifications: A Constantly Evolving Process

**DOI:** 10.3390/epigenomes4040026

**Published:** 2020-11-23

**Authors:** Annalisa Fico, Luciano Di Croce, Maria R. Matarazzo

**Affiliations:** 1 Institute of Genetics and Biophysics ‘Adriano Buzzati-Traverso’, CNR, 80131 Naples, Italy; maria.matarazzo@igb.cnr.it; 2 Stem Cell Fate Laboratory, Institute of Genetics and Biophysics ‘A. Buzzati-Traverso’, CNR, 80131 Naples, Italy; 3 Centre for Genomic Regulation (CRG), Barcelona Institute of Science and Technology (BIST), Dr. Aiguader 88, 08003 Barcelona, Spain; Luciano.DiCroce@crg.eu; 4 Universitat Pompeu Fabra (UPF), 08003 Barcelona, Spain; 5 ICREA, Pg. Lluis Companys 23, 08010 Barcelona, Spain

The epigenome refers to the entirety of DNA methylations, histone modifications, nucleosome occupancy, and coding and non-coding RNAs (and their modifications) in different cell types. These modifications can result in changes to the structure of chromatin as well as changes to the function of the genome. Similarly, the dynamic RNA modifications, which are termed RNA epigenetics or epitranscriptome, include all the post-transcriptional modifications in coding and non-coding RNAs, rRNAs [1,2], and tRNAs in a cell. In this Special Issue, Damien Hermand nicely describes the singular diversity of possible chemical modifications of tRNAs and how they are crucial to striking a balance between translational efficiency and fidelity. The importance of these modifications is also supported by their remarkable conservation from prokaryotes to eukaryotes. tRNA modifiers include the Elongator complex, which has been shown to be involved in a biologically relevant epitranscriptomic pathway targeting the wobble uridine of several tRNAs [3].

The dynamic interplay of DNA and RNA modifications is of crucial significance in orchestrating numerous biological processes in many organisms during early development and cell fate specification. An example is reported by Lasman and colleagues, who clearly describe the importance of m6A modification in stem cell differentiation and gametogenesis, both in males and females. In particular, pluripotent stem cells require m6A modifications to exit from the naïve state in response to differentiation stimuli. Moreover, lack of m6A deposition impairs sperm and oocyte maturation, resulting in reduced fertility [4].

It is clear that epitranscriptomics, like epigenetics, favors combinatorial plasticity supplied by molecules, such as non-coding RNAs and metabolites, that cooperate with protein complexes that function as writers, erasers, and readers of DNA and RNA modifications. Due to their multifaceted nature, long non-coding (lnc) RNAs directly interact with these modifying enzymes, thereby shaping the chromatin state, as described by Fiorenzano and colleagues [5]. They act as guides or dynamic scaffolds by bringing effector proteins together in a spatiotemporal fashion. The ultraconserved lncRNA T-UCstem1 was recently determined to be able to recruit and stabilize the PRC2 complex at chromatin, thereby determining the proper H3K4me3/H3K27me3 ratio at bivalent chromatin domains. T-UCstem1 activity is crucial in preserving transcription dynamics and maintaining embryonic stem cell (ESC) self-renewal [6].

Recently, several studies have reported that the lncRNAs themselves could be modified. Increasing evidence supports that m6A modification of lncRNAs participates in gene regulation via several mechanisms. Briefly, m6A modification of lncRNAs regulates their stability, as shown for FAM225A, and determines their subcellular distribution, through interactions with other lncRNAs, miRNAs, mRNAs, and/or proteins, as in case of the RP11 transcript. Moreover, m6A modification of MALAT1 lncRNA changes its structure by affecting its interaction with protein partners. A very well studied example is the *Xist* transcript, which mediates gene transcription repression on the X chromosome when it has proper m6A modifications. 

However, there are still very few studies on m6A modification of lncRNA, and further exploration of the molecules involved in modification and recognition of the m6A lncRNAs is needed. Additionally, it would be of great interest to understand whether there are differences (and if so, what they are) between m6A modifications on mRNAs or lncRNAs [7].

Another category of key cofactors that modulate the activity of many chromatin-modifying enzymes is metabolite intermediaries, as extensively described by D’Aniello and colleagues in this Special Issue [8]. The ability of metabolites, including acetyl-CoA, S-adenosylmethionine (SAM), alpha-ketoglutarate (αKG), and vitamin C (vitC), to influence global levels of DNA and histone methylation is an increasingly well known phenomenon [9]. Indeed, by modulating the chromatin structure in the nucleus, these metabolites can play a direct role in shaping gene expression programs. The most striking example is the reciprocal counteracting effect of vitC and the non-essential amino acid l-proline (l-Pro) on the JumonjiC domain–containing histone demethylases (JmJC) and the ten–eleven translocation (Tet) DNA demethylases. Vitamin C is a cofactor/enhancer of these two vitC-αKG-dependent dioxygenases that erase DNA and histone methylation; however, it also favors the activity of another dioxygenase, the prolyl-hydroxylase (P4h), which uses l-Pro for collagen synthesis. Therefore, an increase of this enzyme activity upon addition of l-Pro reduces the levels of vitC available for the JmjC and Tet enzymes, which are in turn inhibited. This results in an abnormal increase of DNA and histone methylation in L-Pro supplemented cells [10].

While the metabolic-epigenetic crosstalk was first studied in the context of DNA and histone covalent modifications, recent studies have shown that metabolic regulation also affects other classes of cofactor-utilizing enzymes, including writers and erasers of RNA modifications. For instance, deposition of m6A by the METTL3/METTL14 complex requires SAM; in contrast, it is erased by the vitC-αKG-dependent dioxygenases FTO and ALKBH5, which (similar to Tet and JmJC) are sensitive to the levels of αKG and vitC in the cell [8]. Notably, it has been reported that METTL16 regulatory activity over the MAT2A transcript via m6A deposition depends on the abundance of SAM. Indeed, in low SAM concentrations, METTL16 can stably interact with the MAT2A transcript but cannot properly methylate it. Lack of m6A methylation increases MAT2A stability, thereby maintaining the cellular SAM homeostasis [11].

Epigenomic and epitranscriptomic remodeling in response to metabolic changes are strictly correlated with the acquisition of cell identities during embryo development. This is the case of the cell transition that occurs when ESCs exit from the naïve state to acquire a primed state of pluripotency, and during cell specification. Whether the metabolic reprogramming is a cause or consequence of these transitions during the cell fate decisions is still unclear and needs to be further explored.

The reversibility of RNA modifications adds a new dimension to the developing picture of post-transcriptional control of gene expression programs. This new dimension must now be integrated into our understanding of transcriptional regulation at the DNA modifications level, to decipher the multi-layered controls of a plethora of biological functions. In this scenario, an important breakthrough recently came from work by Li et al. [12], which shows that co-transcriptional m6A deposition of nascent mRNA directly regulates the levels of dimethylated H3K9 (H3K9me2) by recruiting the demethylase KDM3B. The resulting H3K9me2 reduction determines a concomitant positive regulation of transcription. This regulatory mechanism is mediated by the nuclear protein YTHDC1, which recognizes m6A and binds the demethylase, thereby acting as a bridge between DNA and RNA modifications [12]. Further studies in the field will shed light on the coordinated activities of epigenetic and epitranscriptomic players, and will provide us with a more comprehensive view of mechanisms governing cell fate decisions.
